# Heart of Darkness

**DOI:** 10.1089/cren.2016.0114

**Published:** 2016-11-01

**Authors:** Peter Alken

**Affiliations:** Department of Urology, Mannheim University Hospital, Mannheim, Germany.

**Keywords:** percutaneous nephrolithotomy, mortality, Heart of Darkness, Joseph Conrad

## Abstract

Significant literature has an impact on the reader. Reading the novella Heart of Darkness by Joseph Conrad as a young boy rose emotions comparable to those I felt when losing a patient after percutaneous nephrolithotomy (PCNL) as a grown up. The case of a 37-year-old woman with bilateral staghorn and a fatal outcome after PCNL is presented and alternatives are discussed.


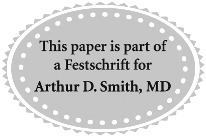

“My purpose was to stroll into the shade for a moment; but no sooner within than it seemed to me I had stepped into a gloomy circle of some Inferno. The rapids were near, and an uninterrupted, uniform, headlong, rushing noise filled the mournful stillness of the grove, where not a breath stirred, not a leaf moved, with a mysterious sound—as though the tearing pace of the launched earth had suddenly become audible.

Black shapes crouched, lay, sat between the trees, leaning against the trunks, clinging to the earth, half coming out, half effaced within the dim light, in all the attitudes of pain, abandonment, and despair. Another mine on the cliff went off, followed by a slight shudder of the soil under my feet. The work was going on. The work! And this was the place where some of the helpers had withdrawn to die.” … “They were dying slowly—it was very clear. They were not enemies, they were not criminals, and they were nothing earthly now,—nothing but black shadows of disease and starvation, lying confusedly in the greenish gloom. Brought from all the recesses of the coast in all the legality of time contracts, lost in uncongenial surroundings, fed on unfamiliar food, they sickened, became inefficient, and were then allowed to crawl away and rest. These moribund shapes were free as air—and nearly as thin.”

Heart of Darkness (1899) Joseph Conrad^1^

I read this novella of Joseph Conrad when I was a young boy. Born in 1942, I had no experience with the cruelties of war or video games. It was for the first time that I saw death approaching and I felt sick and helpless. This feeling reappeared when we treated a patient who died in our department in 1991 and I still remember it. She was the first patient whom I saw dying after percutaneous nephrolithotomy (PCNL), which I had started to do in 1976.

## Case Report

A female 37-year-old patient was admitted to our Department in January 1991. A left complete and a right partial staghorn stone had been diagnosed 8 years before. She had not wanted therapy. In May and June 1990, she had had pneumonia and she had lost 10 kg weight in the last 6 months. She was cachectic with a body mass index (BMI) of 15.8 (159 cm, 40 kg). She had no flank pain, but 39.6°C fever. Urine showed all signs of urinary tract infection (UTI) and the urine culture was positive for *Escherichia coli* with resistance against 8 of 16 tested antibiotics. The white blood count was 11.400 and she was anemic with an Hb of 8.9 g/dL. Her creatinine was 1.5 mg% (0.6–1.3 mg%). Actual plain film and CT showed bilateral complete staghorn stones. There was no visible dye excretion on the left side in the external intravenous urography, and sonography showed hydronephrosis of the left kidney with nearly no parenchyma. In the split isotope function test from June 1990, the left kidney had only 26% function and in January 1991, it was only 13%.

Her temperature came gradually down to below 37°C during 11 days of appropriate antibiosis, the Hb was improved with transfusions, and the creatinine was 1.2 mg%.

On Monday, January 21, 1991, after 2 days without fever, right PCNL was done by an experienced associate: puncture of the lower right calix, dilation to 26F, Amplatz sheath, ultrasound disintegration of the lower caliceal extension and the pelvic portion of the struvite staghorn stone. The middle and upper caliceal extensions were left for secondary extracorporeal shockwave lithotripsy at the end of that week, which was the strategy at that time. There was venous bleeding upon removal of the Amplatz sheath, which stopped after placement of a 22F balloon nephrostomy. The patient was extubated, transferred to the post-acute care unit, and from there during the night to the intensive care unit. The next day, she was reintubated because of respiratory problems. The right kidney functioned well with a urine production of between 100 and 200 mL/hour, while the left kidney produced between 0 and 30 mL/hour. Despite appropriate therapy, her condition worsened and the right kidney, which was thought to be a persistent septic focus, was removed on January 26th. She died on February 1, 1991 due to multiple organ dysfunction.

I do not remember how we discussed the case in our 6-month fatality conference. When you lose a patient, you start thinking about what you could have done different. Your thoughts are orientated toward your past experience and to other urologists' thoughts published in the literature.

What was the cause and what could alternative options have been or be in a similar case?

### The risk of serious PCNL complications

To be able to predict complication of PCNL, Moreno-Palacios et al.^2^ designed an index based on an analysis of 354 PCNLs with four cases of (Clavien 5) deaths and four cases of (Clavien 4) sepsis. A high Charlson comorbidity index (CCI) was most important, predicting a Clavien ≥3 grade complication.

Our patient was at least among the moderate risk group, but the risk cannot be calculated as a low BMI of 15.8 does not fit into the CCI. Would it help to know the risk? Would palliative care have been an option?

### Preliminary drainage

Benson et al.^3^ reported on a special series of 219 patients treated between 2007 and 2012. Nearly, a third of them had lower urinary tract reconstruction or neurogenic bladder, which are known to have a complicated course after PCNL.

Group 1 patients had preprocedural nephrostomy for 19.6 days and antibiosis for an average of 13.7 days, while group 2 had 6.8 days of preoperative antibiosis and access established on the day of surgery.

None of the patients with preprocedural nephrostomy had post-PCNL sepsis and drainage was the only factor preventing it. Two third of the patients with prolonged antibiosis and nearly 3 weeks of nephrostomy drainage still had positive stone cultures. This well-known problem to sterilize the upper urinary tract is difficult if not impossible to manage.^4^

In a similar series with neuromuscular disorders, preliminary drainage between 1 and 62 days, but not prolonged antibiosis, reduced the incidence of bacteremia/sepsis from 5/19 to 0/16.^5^

Reports dealing with aspiration of purulent urine at the time of the puncture saw similar frequencies of septic complications with or without temporary percutaneous drainage and advocated same session PCNL when the patients had no other signs of infection preoperatively.^6,7^

Many other reports on the benefit of decompression of the upper urinary tract deal with obstruction due to ureteral stones and already manifested systemic inflammatory response syndrome (SIRS)/sepsis,^8,9^ do not add to a better understanding of our case.

Preliminary urinary diversion by uni- or bilateral percutaneous nephrostomy would have been an option. My personal experience is that even the puncture to place a small emergency nephrostomy tube for drainage only, sometimes provokes bouts of fever. The drainage, especially for complete staghorn stones, is rarely complete and there are areas left without drainage, without the possibility to recognize these areas and change the conditions.^7^

We could have tried this approach or maybe we should have tried it.

### Staged PCNL procedure

Zhao et al.^10^ looked at the advantage of a staged PCNL procedure. Patients were treated either in planned two sessions (group 1) or in one intended session (group 2) between 2011 and 2013. Patients with UTI were treated with antibiotics for 3–5 days until the culture turned negative. A second stage was done after 3–5 days. There were ∼50% complete stags in both groups and 30% had UTI and infection stones. The results favored the planned two sessions, with SIRS or septic shock happening in 11 (7.5%) group 1 and 22 (15.8%) group 2 patients. Of those patients with infection stones, 22% of group 1 and 41% of group 2 had infection-related complications.

Our patient was treated in a planned two-session procedure.

### Open surgery, laparoscopy

Al-Kohlany et al.^11^ were the first to publish a prospective randomized controlled study comparing a temporary series (2001–2003) of 79 patients and 88 complete staghorn stones treated by open surgery (OS) *vs* PCNL. Unusually, few patients, 15/88 had UTI and only 5 of the 50 stones analyzed were composed of struvite. The intra- and postoperative complication rate of 15/43 for PCNL and 31/45 for OS favored PCNL. Postoperative sepsis was equally distributed with three cases in each group.

Lunardi et al.^12^ did 26 anatrophic nephrolithotomies between 2005 and 2013. Half of the patients had preoperative UTI and only two cases of pyelonephritis were among the few complications. A meta-analysis on PCNL and laparoscopic surgery (LS) for pelvic stones showed principal advantages for the latter with a lower incidence of bleeding, less postoperative fever, and a higher stone-free rate, but the analysis was not focused on staghorn stones.^13^

A more recent publication by Aminsharifi et al.^14^ compared 45 adults who underwent PCNL,^16^ OS,^14^ or LS^15^ between 2010 and 2015.

Active UTI was an exclusion criterion and all patients had antibiotic treatment 1 day before surgery, “so that sterility of the urine was ensured before the operation.” Only 11 patients had struvite stones. For PCNL, they used a 30F Amplatz sheath. Anatrophic nephrolithotomy was used in all other patients.

Only a few patients needed transfusion (three OS, two PCNL, and one LS) and there were no postoperative infectious complications. A significant reduction of renal function in all groups was most pronounced in the open group from 42% to 33%. In the lap-series of Pastore et al.,^15^ all nine patients had a negative preoperative urine culture and received a single dose of broad-spectrum antibiosis. All nine patients were PCNL failures and none of them had struvite stones. After laparoscopic pyelolithotomy and laser disintegration of the peripheral stones through a flexible scope, there were no complications and only one residual stone of 9 mm.

In 1991, OS could have been an option, but it was and is not stylish; laparoscopy was and probably would not be an option today.

### Mortality

Mortality after PCNL is rare. Unsal et al.^16^ saw three mortalities in their multicenter report on 1406 procedures. Desai reported no mortality in their 773 cases of PCNL for staghorn stones^17^ and in the Croes report with 5335 cases, of which 1466 (27.5%) had staghorn stones, mortality was not mentioned.^18^

The above patient is the only fatality in our PCNL report with a mortality rate of 0.3%^19^ and is the only patient I have ever seen dying after PCNL.

Mortality is rarely presented in more than a few words in the urological literature and not as a major topic, especially not in urolithiasis articles. A rare and laudable exception is the article “Mortality and flexible ureteroscopy: analysis of six cases” by Cindolo et al.^20^ However, mortality is not infrequent. A nationwide U.S. sample showed 46,354 patients being hospitalized in 2009 with infected urolithiasis. Nearly 4000 and 1500 had associated sepsis and severe sepsis, respectively. Nine hundred twenty-seven patients had died in 2009 compared to 592 in 1999. The percentage of sepsis or severe sepsis in patients with kidney stones is less than half of those with ureteral stones, but concerning mortality, the percentage is identical.^21^ Probably 1000 people die each year in the United States from infected urolithiasis, but may be only a handful make into a publication.

So is it difficult to learn from our fatal complications or is it impossible because death will come anyway?

We cannot learn a special procedure or a technical detail from these rare cases, but may be a strategy. It is the physician who finds himself in an emergency situation of a personal relationship. The question is not how to remove the stone or how to find a solution, which is focused on the urological problem, but to get access to a human being. It is an emergency situation not only for the patient but also for the surgeon, because he will never have a chance to read what will happen. Other qualities than those of an urologists are required.

“The steamer toiled along slowly on the edge of a black and incomprehensible frenzy. The prehistoric man was cursing us, praying to us, welcoming us—who could tell? We were cut off from the comprehension of our surroundings; we glided past like phantoms, wondering and secretly appalled, as sane men would be before an enthusiastic outbreak in a madhouse. We could not understand, because we were too far and could not remember, because we were traveling in the night of first ages, of those ages that are gone, leaving hardly a sign—and no memories.

The earth seemed unearthly. We are accustomed to look upon the shackled form of a conquered monster, but there—there you could look at a thing monstrous and free. It was unearthly, and the men were—No, they were not inhuman. Well, you know, that was the worst of it—this suspicion of their not being inhuman. It would come slowly to one. They howled, and leaped, and spun, and made horrid faces; but what thrilled you was just the thought of their humanity—like yours—the thought of your remote kinship with this wild and passionate uproar. Ugly, yes, it was ugly enough; but if you were man enough you would admit to yourself that here was in you just the faintest trace of a response to the terrible frankness of that noise, a dim suspicion of there being a meaning in it which you—you so remote from the night of first ages—could comprehend. And why not? The mind of man is capable of anything—because everything is in it, all the past as well as all the future.”^1^

Heart of darkness is still in the board's and in the reader's list of the 100 best novels. However, it has also been criticized to promote prejudice against Africa. I have read the novella again as a grown up and for me, it is a perfect example for the different views on reality and for the forces of fate—black or white.

## Abbreviations Used

BMI: body mass index
CCI: Charlson Comorbidity Index
CT: computed tomography
LS: laparoscopic surgery
OS: open surgery
PCNL: percutaneous nephrolithotomy
SIRS: systemic inflammatory response syndrome
UTI: urinary tract infection
